# Morphological and radiative characteristics of soot aggregates: Experimental and numerical research

**DOI:** 10.1038/s41598-019-57045-y

**Published:** 2020-01-15

**Authors:** Ezgi Sutcu, Nimeti Doner, Fengshan Liu, Umran Ercetin, Fatih Sen, Jérôme Yon, Jose Morán, Andrés Fuentes

**Affiliations:** 1 Dumlupinar University, Engineering Faculty, Mechanical Engineering Department, 43270 Kutahya, Turkey; 2 Gazi University, Engineering Faculty, Mechanical Engineering Department, 06570 Ankara, Turkey; 3 Black Carbon Metrology, Measurement Science and Standards, National Research Council, Ottawa, Ontario, K1A 0R6 Canada; 4 Sen Research Group, Biochemistry Department, Dumlupinar University, 43270 Kutahya, Turkey; 5 Normandie Univ, INSA Rouen, UNIROUEN, CNRS, CORIA, 76000 Rouen, France; 6 Departamento de Industrias, Universidad Técnica Federico Santa María, Av. España 1680, Casilla, 110-V Valparaíso, Chile

**Keywords:** Climate change, Engineering

## Abstract

The study is aimed at investigating the radiative properties of soot aggregates at determined morphological features using both experimental and numerical methods. Soot aggregates collected from air monitoring stations in different locations were examined. The locations were divided into three groups. The first group (Case 1) included the coastal and industrial zone; the second group (Case 2) consisted of small and large cities; and the third group (Case 3) included areas in the neighbourhood of thermal power plants. The absorbance measurements of the soot aggregates were conducted in the visible and near-infrared spectra, and in the wavelength range of 2 μm-20 μm. The samples were characterised by scanning electron microscopy (SEM), and their radiative properties were assessed using the discrete dipole approximation (DDA) for numerically generated fractal aggregates with two popular refractive indices of *m* = 1.60 + 0.60*i* and *m* = 1.90 + 0.75*i*. Calculations were conducted for primary particles in point-contact, with 20% overlapping and with a coating (50% and 80%) in the wavelength range of 0.4–1.064 μm. The largest measured absorbance values in both the winter and summer seasons were found in the cities in Case 1, and the *x*-ray diffraction (XRD) phases of the samples were also presented. The radiative properties of the aggregates, i.e., *D*_*f*_ = 1.78 and *k*_*f*_ = 2.0 representing Case 3, were close to those of aggregates with *D*_*f*_ = 2.1 and *k*_*f*_ = 2.35 representing Case 1 in the investigated wavelength range. The calculated radiative properties and the experimental absorbance measurements for point-contact and overlapping situations showed the same trend in the examined wavelengths. The absorbance properties of the samples of coastal and industrial zones were distinctively higher than others in the wavelength range of 2 μm-20 μm which could be attributed to the PAH effects.

## Introduction

The effects of the components and morphology of soot aggregates on health and climate are known^1–6^, and the effect of combustion conditions on the photochemical properties of soot has also been investigated. Aerosols, such as organic and black carbons, sulphates, nitrates, ammonium, desert dust, and sea salt, which are found in the troposphere, are classified as absorbing (weakly or strongly) and non-absorbing^7,8^. To examine the effects of soot, regional observation centres^9,10^ or satellite lidar such as CALIPSO^11,12^ have been used. Additionally, several programs such as OPAC^13^, AERONET^14–16^, GISS^17,18^, and MOPSMAP^19^ have been completed, and the sources of aerosols have been investigated^20–23^. As a result of the fact that rapidly developing countries such as China and India are in Asia, many studies have been performed on the effects of the radiative properties of soot aggregates on climate change in Asia^24–31^. In one of these studies, Ramanathan *et al*. analysed the effects of changes in air temperature over the Himalayas by examining samples collected via eighteen flights of unmanned aircraft in Asia^27^. They showed the effects of black carbon particles on climate change are almost the same as those of greenhouse gases.

Soot aggregates produced by the incomplete combustion of fossil fuels consist of carbonaceous particles and volatile species, depending on the fuel and/or combustion process^32^. When soot aggregates are transported in the atmosphere, they undergo morphological transformations as a result of the aging process. In the aging process, soot particles interact with other aerosols, such as sulphate, nitrate, and organic matter in the atmosphere, and thereby acquire a coating. Additionally, their morphology is affected by temperature changes and humidity in the atmosphere^33,34^. The formation and growth of soot aggregates during incomplete combustion are attributed to polycyclic aromatic hydrocarbons (PAHs) and small hydrocarbon molecules, which appear in soot particles because of surface interactions^35^. Soot aggregate coating with organic materials during aging leads to enhanced absorption and scattering properties^36^. Detailed explanations of the morphological properties and compositions of soot aggregates can be found in the literature^37,38^.

The present work was aimed at investigating the optical properties of the soot aggregates for the determined morphological features according to their location and industrial conditions (i.e. intensive industrial zones, thermal power plant neighbouring and city center), as well as chemical contents such as carbon, silicon, potassium, and sulphate. To this end, the study was designed as follows. The collected samples and the locations and average periodic PM_10_ measurement results are explained in Section 2.1. The absorbance measurements and their results are given in Section 2.2 and the explanation of morphological properties such as overlap, necking, and coating are provided in Section 2.3. The details of the discrete dipole approximation (DDA) method are presented in Section 2.4. The radiative properties of soot aggregates were evaluated experimentally by performing absorbance measurements and numerically using the DDA. The absorbance measurements of the samples in the wavelength range of 2 μm to 20 μm were studied to demonstrate the presence of PAH and peroxyacetyl nitrate (PAN). According to the authors, such an examination on soot aggregates was made for the first time. Additionally, the samples were morphologically examined by assessing more than 250 SEM images. The soot morphological parameters inferred from SEM image analysis were used to generate numerical aggregates for DDA calculations in the UV to the infrared spectral range. The location effects and emission/fuel properties of the investigated cases were also presented in Section 3 by the compositions (EDX), XRD phase analyses.

## Sampling and Analysis

### Sample collection

**Figure 1:**
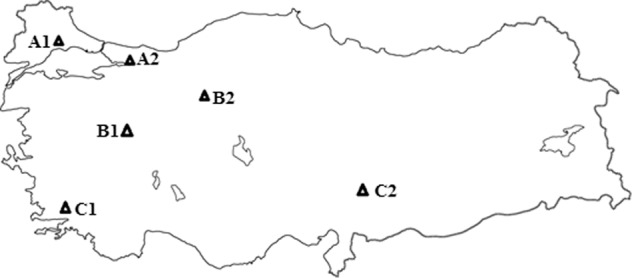
The locations of the cities where the soot samples were collected.

**Figure 2:**
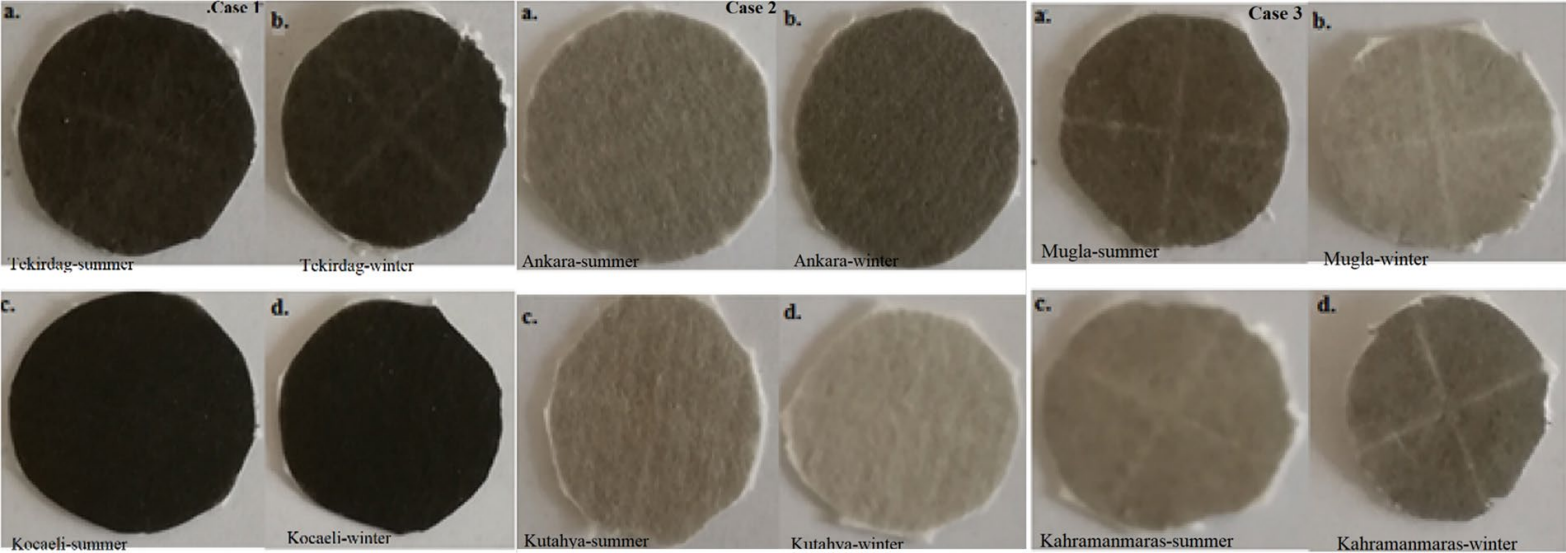
The images of the collected samples.

Over 1000 approximately one or two hour aged samples, were obtained from air monitoring stations in the city centers of the divided three groups according to location and industrial features in Turkey. The samples were taken many times in both the winter and summer in 2017. The geographical locations of the cities where the samples were obtained and the images of some samples are shown in Figs. 1 and 2, respectively. Here, as well as in the representative SEM images and EDX results, the city names are coded for simplicity (A1: Tekirdag, A2: Kocaeli, B1: Kutahya, B2: Ankara, C1: Mugla, C2: Kahramanmaras). Case 1 (Tekirdag 27°52ʹ N, 40°98ʹ E, Kocaeli 29°88ʹ N, 40°85ʹ E) represents coastal and industrial areas with approximately equal populations. Many industrial factories, such as machine manufacturing, automotive, refinery, paper, chemical, and petrochemistry, are located in Kocaeli. Case 2 (Kutahya, Ankara) represents cities with a continental climate but different populations. Ankara (32°52ʹ N, 39°56ʹ E) in Case 2 is the capital of Turkey; with a population of 5.25 million, it is the most crowded city among all the considered cities. Case 3 (Mugla 28°36ʹ N, 37°22ʹ E, Kahramanmaras 36°94ʹ N, 37°59ʹ E) represents cities around thermal power plants; their populations and climates are approximately the same. In the air monitoring stations in the cities, measurements of sulphur dioxide, carbon monoxide, particulate matter (PM_2.5_ and PM_10_), and meteorological conditions (humidity, pressure, temperature, wind speed and direction) were made. Based on the records of the air monitoring stations where they measured PM_10_ captured by the attenuation of beta rays, the PM_10_ content of the investigated Kocaeli winter sample was obtained at an average value of 152.67 μg/m^3^, and the summer sample was at an average value of 52.25 μg/m^3^. For Tekirdag in Case 1, while the PM_10_ content of the winter sample was at an average value of 124 μg/m^3^, the summer sample was at an average value of 45 μg/m^3^. In Case 3, the PM_10_ content in the Mugla winter sample was obtained at an average value of 128 μg/m^3^, and the summer sample was at an average value of 72 μg/m^3^. The standard value of PM_10_ is 50 μg/m^3^.

### Absorbance measurements

The attenuation of monochromatic, collimated light passing through the medium of the collection of particles is modeled by the Beer-Lambert law: ${I}_{x}={I}_{0}{e}^{(-\sigma x)}$, where *I*_*x*_ is collimated light intensity at point *x*, *I*_0_ is initial intensity at *x* = 0, and *σ* (m^−1^) is the particle extinction coefficient of the medium. Here, *σx* is referred as the optical depth of the collection of particles and the transmission of light (*I*_*x*_/*I*_0_) is a function of both the distance travelled by the light and the extinction coefficient of the particle-laden aerosol^39^.

**Figure 3:**
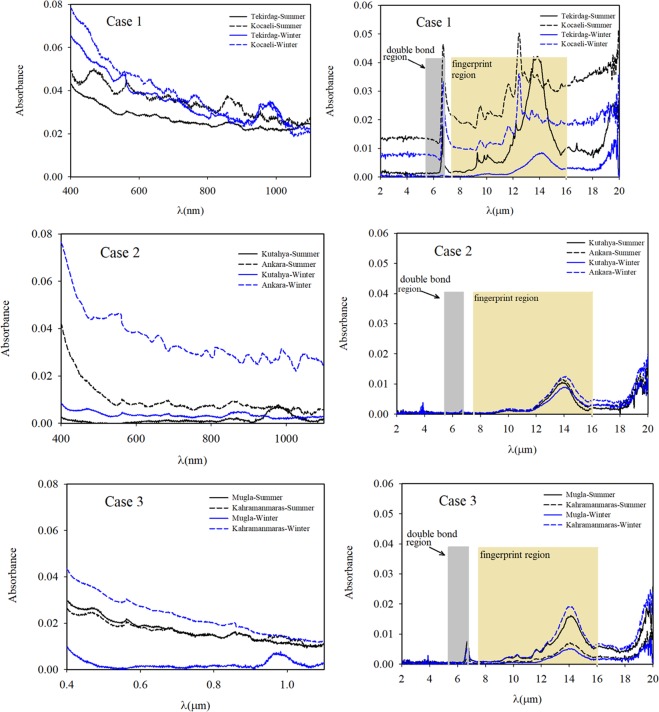
(**a**) Absorbance measurement results for Cases 1, 2, and 3 in the visible to near IR spectrum. **(b)** Absorbance measurement results for Cases 1, 2, and 3 at a wavelength range of 2 μm to 20 μm.

In our study, the absorbance measurements were performed with two spectrophotometers using 250 samples. Firstly, for the 50 samples, the PerkinElmer Lambda 750 spectrophotometer was used for absorbance measurements in the wavelength range of UV to near IR at room temperature. Solutions (20 mL, containing ethanol) of the samples were prepared using an ultrasonic tip sonicator (Wisd) for 30 minutes. The solutions were then passed through a membrane to obtain a homogeneous particle size distribution. Then, the reference measurements for ethanol were performed in the device, and the prepared samples were measured. These measurements were performed at least three times with good repeatability. Secondly, for around 200 samples, the PerkinElmer Spectrum Two spectrophotometer was used for absorbance measurements in the wavelength range of 2 μm to 20 μm at room temperature. For these measurements, the measuring tip of the device made measurements of the absorbance by touching the sample at a point. The clean filter paper was first measured, and subsequently, the absorbance measurements of the air-absorbed filter papers coated with soot were performed. In these measurements, the various surfaces (sides and centre) of the soot-coated filter papers were first investigated. Afterwards, as almost the same values were obtained, the measurements were made in the middle of the filter paper coated with soot. The measurements were conducted at least three times independently with good repeatability. Figure 3(a,b) present the absorbance measurements of the samples obtained for Cases 1–3 in the UV to near IR spectrum and the wavelength range of 2 μm to 20 μm, respectively.

When the absorbance measurements of all cases were evaluated at the wavelength range of 0.4–1.1 µm for both the winter and summer seasons, it was found that the absorbance results of Case 1 and Ankara-Winter in Case 2 were high at short wavelengths. The highest absorbance values in the visible spectral range were found at 0.08 for the Kocaeli-Winter sample in Case 1 and the Ankara-Winter sample in Case 2. Figure 3 shows that the Tekirdag samples had slightly lower absorption values than the Kocaeli samples. As shown in Fig. 3(a), the absorbance values of Kahramanmaras-Winter in Case 3 were as high as the absorbance value of Tekirdag-Summer in the industrial zone, and the absorbance values of Mugla-Winter in Case 3 was the lowest compared to all the other measurements in Case 3. However, the absorbance values of Mugla-Summer were higher than the Kahramanmaras-Summer values. This is thought to be because of vehicular emissions and a seasonal effect as Mugla is in a popular tourist region in the summer.

As mentioned in earlier studies^40–43^, the double-bond structures are revealed in the wavelength range of ~5.5–6.3 μm (double bond region) while the one-bond structures are observed in the wavelength range of ~7.1–16 μm (fingerprint region) with oxygen, nitrogen and carbon. Although aromatic hydrocarbons and PAN are usually together, the absorbance spectrum of PAH is in the fingerprint region^43^ and the absorbance spectrum of PAN^40^ is in the double bond region. As a result of strong structural bonds in PAH and PAN, their absorbance properties can be detected at long wavelengths. Figure 3(b) shows these the wavelength ranges and the absorbance measurement results of the samples. In other words, soot generally consists of structures containing several functional groups, such as C=O and C-O in graphene layers. When soot is dispersed into the atmosphere, the particles are subject to the aging process, in which carbonyl C=O and ether C-O groups on the surface of soot increase significantly. SO_2_ was oxidized to sulphate species, evidenced by the rapid increase in IR spectral density in the 7.54 μm-10.1 μm region. Moreover, sulphate production on soot particles in an oxygen-rich environment was significantly higher than the results in the oxygen-free medium. As a result, the soot had a significant catalytic effect for SO_2_ oxidation and sulphate formation with the aid of O_2_. In Kocaeli in Case 1, three peaks were observed at the wavelength range of the fingerprint region. These peaks are thought to result from PAH molecules. In Kahramanmaras-winter and Mugla-summer samples in Case 3, the three peaks are believed to be from PAH molecules and one peak from PAN molecules at the investigated wavelengths.

### Overlapping, necking and coating in the aging period

Overlapping between neighboring primary particles, which is quantified by *C*_*ov*_, a morphological parameter^44^, can occur during the aggregate growth process. The degree of overlapping is used to describe the penetration of primary particles into each other and is formulated by *C*_*ov*_ = [(*r*_1_ + *r*_2_) − *d*_12_]/(*r*_1_ + *r*_2_), where *r*_1_ and *r*_2_ are radii of two primary particles and *d*_12_ is the Euclidean distance between the centers of both primary particles^45–47^. Primary particle overlapping affects *k*_*f*_ and *R*_*g*_ in such a way that *k*_*f0*_ = *k*_*f*_ *exp*[2.2 *C*_*ov*_] and *R*_*1g*_ = *R*_*g0*_(1−*C*_*ov*_), as previously described in ref. ^48^. Here, *R*_*g0*_ and *R*_*1g*_ are the radius of gyration without and with primary particle overlapping, respectively, and *k*_*f0*_ and *k*_*f*_ are the pre-factors of the aggregate without and with primary particle overlapping, respectively. Necking represents the physical connection phenomenon between primary particles^49^ and is used in the simulation of another aspect of aggregate morphology^50^. When soot particles undergo the aging process under atmospheric conditions, the particles interact and mix with non-refractory materials, including sulphate, nitrate, and organic carbon. Mixing can be both internal and external. These interactions form a coating on the soot particle surface to a certain thickness. The effect of the coating is evaluated using different refractive indices and dipoles of the coating. Therefore, in order to consider this interaction through realistic soot morphology, the algorithm of adding dipoles to represent the coating on soot aggregates developed by Yon *et al*.^47^ was used in this study. Using the desired overlapping and necking values in the program, the numerically generated soot aggregates can be coated at specified percentage values.

### Discrete dipole approximation (DDA)

DDA is a powerful numerical method for calculating the scattering and absorption of arbitrary geometry, which can be solved using Maxwell’s equations. In the DDA method, the DDSCAT code developed by Draine and Flatau^51^ is used. The method discretizes particles or clusters of any shape and composition in the form of a lattice structure and polarizable points (dipoles). In order to accurately model the radiative properties of the particles under consideration, it is desirable that the lattice length between the dipoles must be small enough to be comparable to the wavelength of light used^52,53^. This condition is limited by using the expression |*m*|*kd* < 1, where *d* is defined as the lattice length, *m* is the refractive index, and *k* is the wavenumber (2π/λ). The number of dipoles required to calculate the radiative properties increases as the size of the cluster increases; moreover, the computing time of the solution of the matrix equations increases with O (*N*^2^). Here *N* represents the total number of dipoles used in the calculation. When the material being examined is strongly absorbing, or a much more accurate solution is required, the desired condition is taken as |*m*|*kd* < 0.5. The effective radius of the aggregates *a*_*eff*_ = [3 *V*/4π]^1/3^, *V* is the total volume of the aggregate at the examined situation, wavelength, the Euler angles (*β*, *Ω*, *Φ*), and the refractive indices are used in the calculations as input data. As the second important parameter for the considered accuracy, the orientation average should be performed over the entire range of Euler angles. In our study, since DDA provides numerically-exact results^54,55^, the method was selected and different numbers of orientations in the calculations were considered, such as 5 × 5 × 5, 10 × 10 × 10, and 15 × 15 × 15, and the results were compared. It was found that the results obtained using 15 × 15 × 15 could be considered independent of orientation averaging; hence, all the results presented in this paper were obtained using 15 × 15 × 15 orientations. In DDSCAT calculations, N_SPHERES and FROM FILE routines are used to examine the radiative properties of aggregates of spherical particles^56^. While the bare aggregates and overlapping situations are calculated by the N_SPHERES routine, the coating aggregates are performed by the FROM FILE routine. In coating calculations, the shape files, which are prepared by the special algorithm, containing dipoles of overlap and necking, were separately prepared. The extinction cross-sectional area is the sum of the absorption and scattering cross-sectional areas (*C*_*ext*_ = *C*_*abs*_ + *C*_*sca*_). The terms *Q*_*ext*_, *Q*_*abs*_, and *Q*_*sca*_ are the efficiency factors of extinction, absorption, and scattering, respectively. The cross-sectional area is *C*_*i*_ = *Q*_*i*_ π${a}_{eff}^{2}$, with subscript *i* representing extinction, absorption, or scattering.

The morphological parameters of soot aggregates such as fractal dimension (*D*_*f*_) and pre-factor (*k*_*f*_) should be defined in order to numerically generate of aggregates. *D*_*f*_ is the most important morphological parameter of aggregates and is independent of *k*_*f*_ which defines open structure (fluffy value) of the fractal aggregates. These parameters are determined by the scaling law using geometric values such as number of particles, average particle diameter, and aggregate size obtained from transmission electron microscopy (TEM) or SEM images^39^. Besides, the parameter *D*_*f*_ is classified according to combustion conditions, the aging process, and different atmospheric conditions. In a recent study by Wang *et al*.^57^, the different mixing structures and morphologies of soot aggregates collected from different locations and combustion sources were investigated using TEM. They reported the *D*_*f*_ values of fresh soot particles of urban and soot aggregates from vehicle emissions are in the range of 1.52–1.94 while the *D*_*f*_ values of embedded soot particles are in the range of 1.90–2.16. The emission sources of soot aggregates are another important parameter related to the soot morphology, and this parameter is investigated using particulate matter (PM_2.5_ and PM_10_) in soot aggregates. Islam *et al*.^58^ studied the content, XRD phases, and absorbance measurements of the PM_10_ particle matter that comes from diesel train emissions and found that the concentrations of PAH in the PM_10_ matter are highly dependent on particulate matter.

## Results and Discussion

**Figure 4:**
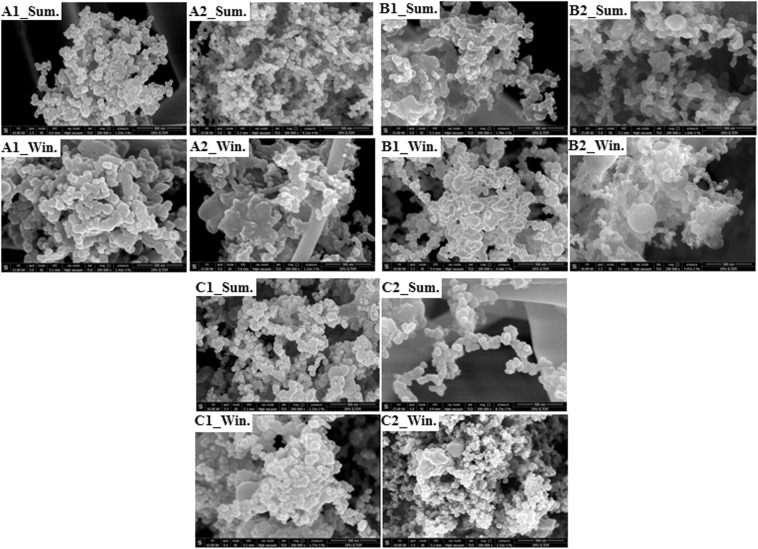
SEM images of the samples.

The morphological properties of the soot aggregates were examined by scanning electron microscopy (SEM; FEI Nova Nano SEM 650). Over 250 SEM images of the samples were examined for the representation of the morphological properties in the aging process. Some of the SEM images in both the winter and summer seasons of the collected soot samples are presented in Fig. 4. In Fig. 4, with a scale bar of 500 nm in all images, it can be seen that all samples have a certain thickness of coating in the winter. The particle size distributions were analysed using the ImageJ program on SEM images. According to the SEM images and ImageJ, the soot aggregates consisted of polydisperse primary particles with an average particle radius of *a = *30 nm and different morphologies. In the calculations, all cases were assumed to have the same polydisperse particle size distribution and the same particle number (*N* = 200); however, they have different fractal dimensions and pre-factors as mentioned above. As the SEM images of the samples taken from the intensive industrial and coastal zone are in compact morphology, the *D*_*f*_ coefficient of Case 1 was appointed as 2.1. As the SEM images of the samples of the other locations are in chain (or lacy) morphology, the *D*_*f*_ coefficients of cases 2 and 3 are the same; however, their pre-factor coefficients were defined differently. Finally, the morphological properties were characterised by *D*_*f*_ = 2.1, *k*_*f*_  = 2.35 for Case 1, *D*_*f*_  = 1.78, *k*_*f*_  = 1.3 for Case 2, and *D*_*f*_  = 1.78, *k*_*f*_  = 2.0 for Case 3. Here, the morphological characterization of cases 1, 2, and 3 was defined according to Zhang *et al*.^21^, Wang *et al*.^57^, Liu and Mishchenko^59^, and Wang *et al*.^60^’s findings, respectively. The soot aggregates were generated using the tunable FracVAL algorithm, which is an improved cluster-cluster aggregation for generating fractal structures formed by polydisperse primary particles^61^.

The refractive index (*m* = *n* + *ki*) of soot aggregates varies with fuel and/or combustion conditions^62,63^. Therefore, we used two typical refractive indices of *m* = 1.60 + 0.60*i* and *m* = 1.90 + 0.75*i* in the analysis of radiative properties of freshly emitted soot aggregates in the considered wavelength range. The former was obtained from Dalzell and Sarofim^64^’s experimental studies, and the latter is the recommended value by Bond and Bergstrom^65^. In evaluating the morphological properties of soot aggregates, China S. *et al*.^66^ and Liu S. *et al*.^67^ classified soot aggregates into three morphologies according to field and laboratory studies such as bare aggregates with point-contact particles, as well as partly and heavily coated aggregates. In this study, all the cases were evaluated in the following three situations: primary particles in point-contact, those with 20% overlapping, and those with a coating to mimic realistic soot aggregates. Here, the first two situations represent bare aggregates, while the coating morphologies (the third situation) represent partially coated and embedded soot, based on refs. ^66,67^ and a recent study by Chakrabarty and Heinson^68^. For the coating situations, based on the results of our previous study^36^, there is some deviation in the results of coatings under 50% in coating thickness for the same overlapping and necking values, and the results of the coating thickness of 80% or more provide almost the same results. Therefore, the coating thickness was taken as 50% and 80%.

**Figure 5:**
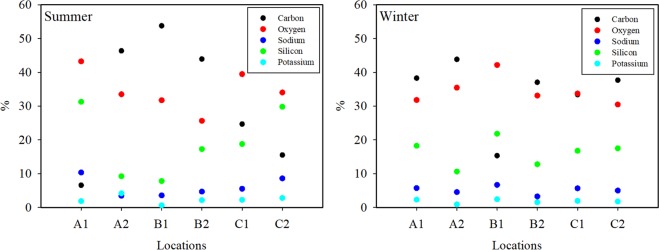
The results of EDX analysis of the samples.

To clarify the differences in the collected soot aggregates, it was necessary to investigate their contents. The chemical compounds of the samples were provided by energy-dispersive *x*-ray spectroscopy (EDX) analysis. The results of the EDX analysis of the samples are shown in Fig. 5. Carbon and oxygen were the primary elements of the samples. According to the EDX results, it can be seen that while the degree of highest carbon emission was in Kutahya in Case 2 in the summer, the lowest carbon emission was in Kahramanmaras in Case 3 and Tekirdag in Case 1. The emitted soot in the cities in Case 1 and Ankara in Case 2 was high carbon soot in the winter. Although the silicon contents of samples from Tekirdag and Kahramanmaras in the summer season were around 30%, they were around 20% in these cities and Kutahya in the winter. It is known that potassium in fine particles is caused by biomass burning, and potassium in large particles originates from dust^69^. In all cities, the potassium content of the samples was found to be less than 5% and originated from biomass burning. Another evaluation feature according to the content is the absorption Ångström exponent (AAE)^59^. The spectral characteristic of particle absorption is formulated by the absorption Ångström exponent (AAE). The AAE calculation can be used to determine whether the source of combustion or elementary carbon is black carbon, according to content analysis and particulate absorption. The AAE values of all the samples were found to be small than unity for both refractive indices assumed in the prediction at a wavelength range of 0.4–1.064 μm. Based on the literature^59^, it can be assumed the samples are black carbon.

**Figure 6:**
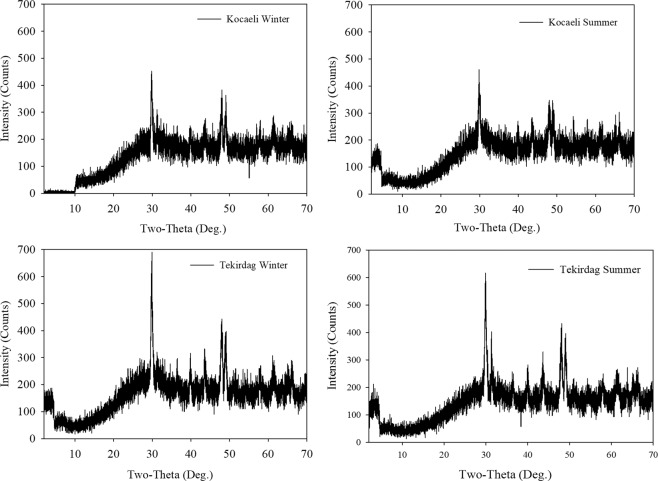
The XRD phases of samples from Case 1.

Figure 6 shows the XRD phases (Rigaku, Model: Miniflex) of soot aggregates in Case 1 for both the summer and winter seasons. The XRD phase analysis shows the mineral phases of soot aggregates. The detailed XRD analysis was also performed by Islam *et al*.^58^ and Dailli *et al*.^70^. As in the absorbance measurement results, although they had the same XRD phases, the XRD phases of the Kocaeli samples were slightly bigger than the patterns of the Tekirdag samples.

Sulphates and nitrates are formed in the atmosphere as a result of the oxidation of SOx and NOx released from combustion devices in industrially-active regions^71,72^. In recent studies, it was determined the production of sulphates and ammonium increases in the daytime and enhances the absorption of black carbon^73^ as a result of a thick coating with abundant sulphate products^74^. Similarly, the PM_10_ values of Kocaeli in both seasons were high in the daytime, and the measured absorption values were also the highest. Additionally, according to NASA^75^, 2017 was the second warmest year in the last 140 years. Therefore, because of the warm weather and based on the abovementioned results of both the EDX and the XRD analysis, we can consider that these results can be attributed to intense traffic emissions and industrial combustion, not from heating emissions.

**Figure 7:**
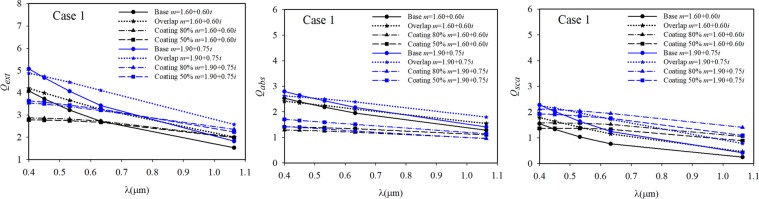
The radiative efficiency factors of Case 1 sample.

**Figure 8:**
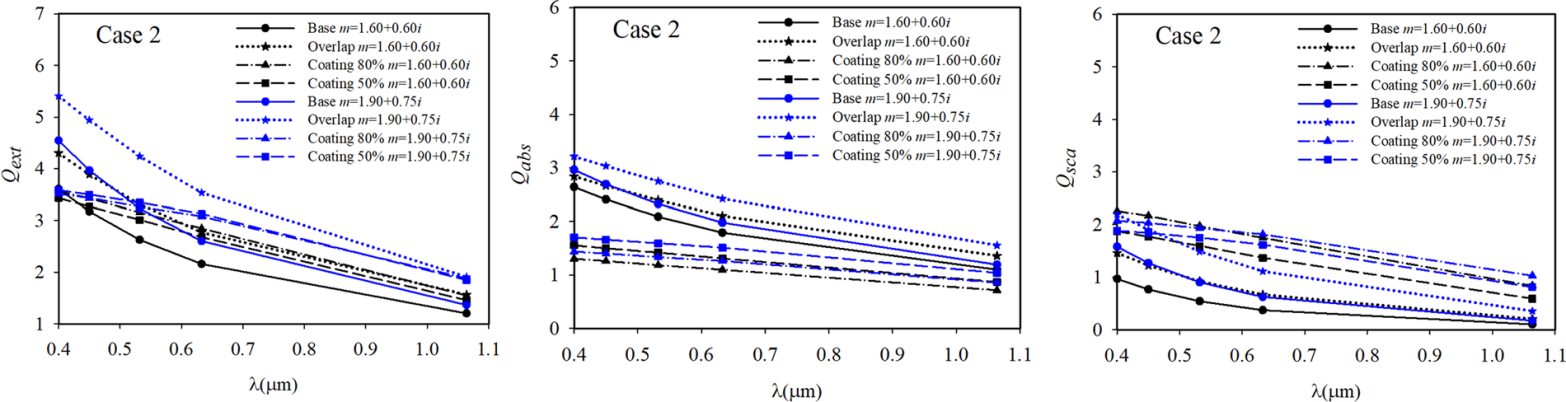
The radiative efficiency factors of Case 2 samples.

**Figure 9:**
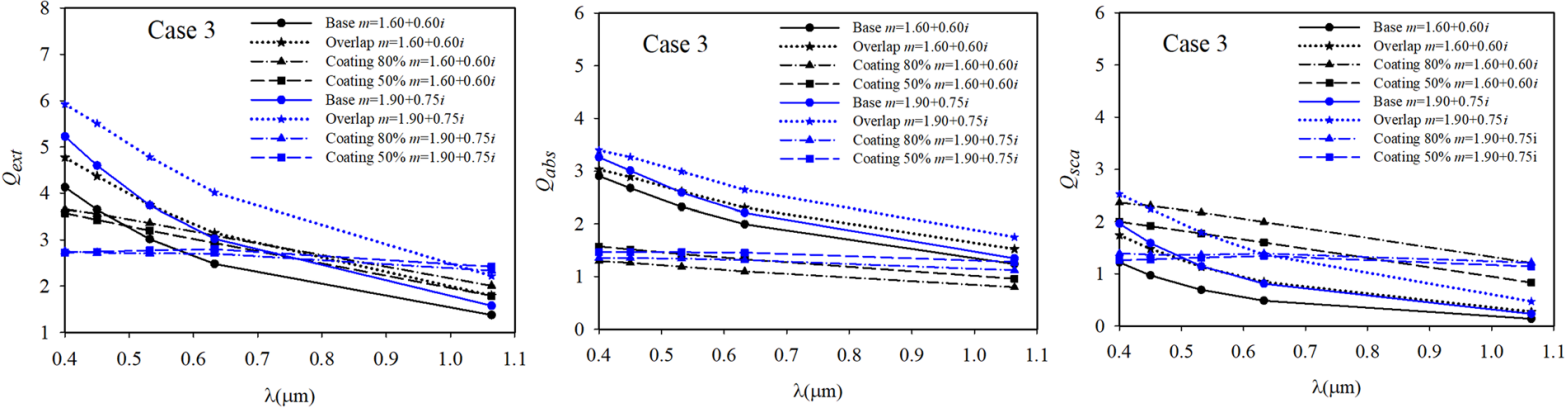
The radiative efficiency factors of Case 3 samples.

The values of volume equivalent radius and the number of dipoles in the DDSCAT calculations for all cases are presented in Table 1. The radiative efficiency factors of cases 1, 2, and 3 in the spectrum of 0.4–1.064 μm are displayed in Figs. 7–9, respectively. The radiative properties of the cases with primary particles in point-contact, with 20% overlapping, and with 50% and 80% coating thickness are shown in these figures. In the cases of coated aggregates, the overlapping and necking were taken as 20% and 0.5, respectively, based on a previous study^36^. The refractive index of the non-absorbing coating material was assumed to be *m* = 1.46.

**Table 1:** The equivalent volume radius (*a*_*eff*_) for all studied cases.

	*a*_*eff*_ [*μ*m]			Number of dipoles		
	Case 1	Case 2	Case 3	Case 1	Case 2	Case 3
Point-contact	0.18364	0.15349	0.17406	353814	81454	216727
20% overlapping	0.22037	0.18371	0.20887	561955	131592	342393
50% coating	0.31884	0.28585	0.33343	316549	389278	418351
80% coating	0.35745	0.33132	0.38943	446120	606320	667014

According to the modelling results shown in Figs. 7–9, an important change in the absorbance values in the wavelength range of 0.4–1.1 μm was observed. Particularly, these changes in absorbance were observed in the short wavelength range. The results obtained with both refractive indices of *m* = 1.60 + 0.60*i* and *m* = 1.90 + 0.75*i* were similar for all the investigated soot morphologies, and it was observed that all the radiative efficiencies decreased with increasing wavelength.

When the results shown in Figs. 7–9 were evaluated according to the two refractive indices, the results obtained with *m* = 1.90 + 0.75*i* for some investigated cases were higher than those with *m* = 1.60 + 0.60*i* at wavelengths above about 632 nm. This observation has been made in previous studies^62,63^. In addition, Morán *et al*.^48^ concluded the multiple internal scattering and the magnitude of this effect are strongly affected by the refractive index. When comparing the results of freshly emitted and overlapping situations, while the extinction, absorption, and scattering efficiencies of Case 1 increase by approximately 13%, 3%, and 36% at a wavelength of 0.532 μm, the radiative properties of Case 3 increase by approximately 24%, 13%, and 64% at the same wavelength, respectively. When the same comparison was made for Case 2, the extinction, absorption, and scattering efficiencies of Case 2 increased by 26%, 15%, and 70%. The relative change was calculated as [(*Q*_*over*_ - *Q*_*bare*_)/*Q*_*bare*_ × 100]. Several studies indicate that an increase in morphological compactness results in an increase in radiative properties^22,23,34,50^. However, it was determined the radiative properties of soot aggregates do not vary according to the compactness but to the volumetric equivalent radius, as mentioned by Luo *et al*.^76^ and Scarnato *et al*.^77^. Although the morphology of Case 1 was more compact than that of Case 3, the absorbance values of Case 1 were lower than those of Case 3 in both the bare and coated situations. The effect of increasing fractal compactness is to decrease absorption cross-sections. However, in the overlapping case, the results of Case 1 were greater than Case 3. This is caused by the multiple scattering effects of primary particles, which can enhance or suppress aggregate absorption. Luo *et al*.^76^ stated the fluffier morphology, which results in a weaker multiple scattering among primary particles, provides more absorbing materials exposed to light.

**Figure 10:**
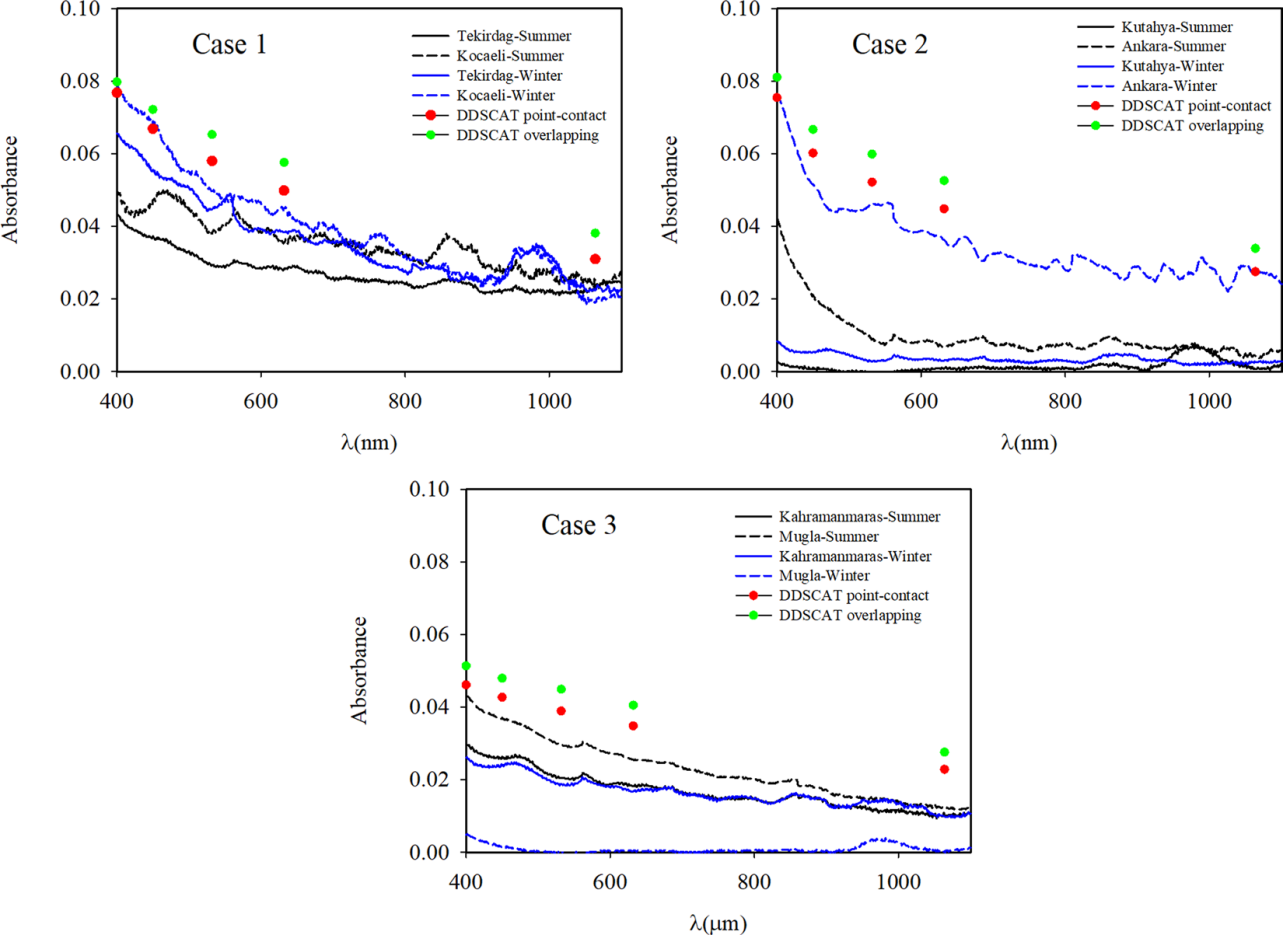
The change trends of calculated and measured absorption results.

When we evaluate the calculated results together with the absorbance measurements in the wavelength range of 0.4–1.1 μm, we need to consider some details. In our absorbance measurements, the particle concentrations of the prepared solutions for the cases were low, and the radiative properties of the soot aggregates inside these solutions were measured. It means, the number of particles or the *a*_*eff*_ values of the measured samples in the experimental studies were small. The samples examined in the DDSCAT calculations have a different *a*_*eff*_. Therefore, the experimental and numerical results should not be compared quantitatively; only the trends in the changes in both cases can be assessed. Similarly, the coating morphologies of the soot aggregates should also not be compared. Figure 10 displays the trends of both results of the measurements and the calculated morphologies are in point-contact and 20% overlapping. In our study, the extinction and absorption efficiency factors showed an approximately 20% decrease in both the experimental and numerical results at short wavelengths. According to the recent study performed by Liu and Mishchenko^59^, the radiative properties of soot aggregates are sensitive to morphology, particle size, sulphate amount, and heterogeneity. The results of our study are consistent with these findings^59^.

## Conclusions

Soot samples collected from different locations in Turkey were analysed by experimental absorbance measurements (FT-IR), content (EDX), and XRD phases. The soot aggregates were numerically generated based on the morphological parameters from SEM images for modelling their radiative properties by DDA. The soot aggregates were modelled about their morphological features such as point-touch, overlapping 20%, and coating thickness 50% and 80% with necking of 0.5 for both refractive indices (*m* = 1.60 + 0.60*i* and *m* = 1.90 + 0.75*i*). PAH and PAN molecule presence was observed by the absorbance measurements in the wavelength range of 2 μm − 20 μm. The samples from Case 1, i.e., a coastal and intensive industrial zone, had the highest measured absorbance values in the wavelength range of 0.4–1.1 μm and had compact morphological features. The lowest absorbance measurement values in the UV-visible spectrum range were seen in the samples from Case 3. As a result, the absorbance values at long wavelengths were smaller than the values in the short wavelength range, except for some seasonal values. According to the DDA results, the radiative properties of Case 3, representing cities close to thermal power plants, which had no compact morphology, were close to the results of Case 1. The radiative properties of the modelled base and overlapping cases showed similar trends to the absorbance measurements.
